# MitoQ Prevents Human Breast Cancer Recurrence and Lung Metastasis in Mice †

**DOI:** 10.3390/cancers14061488

**Published:** 2022-03-15

**Authors:** Tania Capeloa, Joanna Krzystyniak, Amanda Canas Rodriguez, Valéry L. Payen, Luca X. Zampieri, Erica Pranzini, Françoise Derouane, Thibaut Vazeille, Caroline Bouzin, François P. Duhoux, Michael P. Murphy, Paolo E. Porporato, Pierre Sonveaux

**Affiliations:** 1 Pole of Pharmacology and Therapeutics, Institut de Recherche Expérimentale et Clinique (IREC), Université Catholique de Louvain (UCLouvain), 1200 Brussels, Belgium; tania.demiranda@uclouvain.be (T.C.); joannakrzystyniak@yahoo.co.uk (J.K.); amandacanasrodriguez@gmail.com (A.C.R.); valerypayen@hotmail.com (V.L.P.); luca.zampieri@uclouvain.be (L.X.Z.); thibaut.vazeille@uclouvain.be (T.V.); 2 Department of Experimental and Clinical Biomedical Sciences Mario Serio, University of Florence, 50134 Firenze, Italy; erica.pranzini@unifi.it; 3 Pole of Medical Imaging, Radiotherapy and Oncology, Institut de Recherche Expérimentale et Clinique (IREC), Université Catholique de Louvain (UCLouvain), 1200 Brussels, Belgium; francoise.derouane@uclouvain.be (F.D.); francois.duhoux@uclouvain.be (F.P.D.); 4 Department of Medical Oncology, Institut Roi Albert II, Cliniques Universitaires Saint-Luc, 1200 Brussels, Belgium; 5 IREC Imaging Platform (2IP), Université Catholique de Louvain (UCLouvain), 1200 Brussels, Belgium; caroline.bouzin@uclouvain.be; 6 MRC Mitochondrial Biology Unit, Department of Medicine, University of Cambridge, Cambridge CB2 0XY, UK; mpm@mrc-mbu.cam.ac.uk; 7 Department of Molecular Biotechnology and Health Science, Molecular Biotechnology Center, University of Turin, 10126 Turin, Italy; paolo.porporato@unito.it

**Keywords:** breast cancer, cancer relapse, metastasis, mitochondria, mitochondrial superoxide, MitoQ, mitochondria-targeted antioxidant, metastasis prevention, translational research

## Abstract

**Simple Summary:**

Entry in the metastatic phase is often devastating for cancer patients. Metastases originate from metastatic progenitor cells that are selected in the primary tumor and which simultaneously possess several phenotypic capabilities, including migration, invasion, and clonogenicity. We previously provided in vitro evidence that these features are collectively enforced by mitochondrial superoxide in a paradigm where mitochondria act as metabolic sensors of the tumor microenvironment and produce subcytotoxic levels of superoxide to prime metastatic progenitor cells. We also showed that these metastatic traits can be collectively countered by MitoQ, a mitochondria-targeted antioxidant that selectively deactivates mitochondrial superoxide. Here, we further establish that MitoQ prevents primary tumor recurrence after surgery, tumor take and metastasis as a whole, notably in a model of human breast cancer in mice. Since MitoQ already successfully passed Phase I clinical trials, our findings support the development of this drug as a preventive treatment against breast cancer metastasis.

**Abstract:**

In oncology, the occurrence of distant metastases often marks the transition from curative to palliative care. Such outcome is highly predictable for breast cancer patients, even if tumors are detected early, and there is no specific treatment to prevent metastasis. Previous observations indicated that cancer cell mitochondria are bioenergetic sensors of the tumor microenvironment that produce superoxide to promote evasion. Here, we tested whether mitochondria-targeted antioxidant MitoQ is capable to prevent metastasis in the MDA-MB-231 model of triple-negative human breast cancer in mice and in the MMTV-PyMT model of spontaneously metastatic mouse breast cancer. At clinically relevant doses, we report that MitoQ not only prevented metastatic take and dissemination, but also local recurrence after surgery. We further provide in vitro evidence that MitoQ does not interfere with conventional chemotherapies used to treat breast cancer patients. Since MitoQ already successfully passed Phase I safety clinical trials, our preclinical data collectively provide a strong incentive to test this drug for the prevention of cancer dissemination and relapse in clinical trials with breast cancer patients.

## 1. Introduction

Metastatic dissemination and primary tumor recurrence are a particularly bad prognosis for breast cancer patients. In particular, secondary tumors are often more resistant to conventional treatments than primary tumors [1], and local treatments are usually not an option for metastatic patients. In addition, immunotherapy using checkpoint inhibitors only generates a modest response in breast cancer [2]. Despite progress for HER2+ breast cancer [3], polymetastatic breast cancer is still a largely incurable disease [4].

Triple-negative breast cancer (TNBC) and human epidermal growth factor receptor 2-positive (HER2+) breast cancer metastasize and relapse earlier than hormone-dependent progesterone receptor-positive (PR+) and estrogen receptor-positive (ER+) breast cancers [5]. However, although dissemination and recurrence are most often predictable and account for a large majority of patient deaths [4,5], there is currently no treatment preventing these events in breast cancer. Treatments primarily targeting the primary tumor can reduce the risk of relapse, especially when a complete pathological response is reached, but they are not preventive. In clinical routine, patients at risk are closely followed according to guidelines in order to initiate treatments for the metastatic disease with minimal delays [6]. In case of relapse, multiple lines of palliative treatment can be proposed sequentially, but options are limited, especially in TNBC. They do not prevent relapse, are most often not curative, but they can extend the lifespan of the patient with a decent quality of life.

Recently, based on the seminal demonstration of Ishikawa et al. that transferring mitochondria from metastatic to nonmetastatic cancer cells could also transfer the metastatic phenotype [7], we started to explore the hypothesis that cancer metastasis is under metabolic control. Using in vitro and in vivo selection procedures to derivate super-invasive and super-metastatic cancer cells from human cervix adenocarcinoma cancer cells and mouse melanoma cells, respectively, as well as experimental manipulations, we established that mitochondria directly contribute to the metastatic process when they produce superoxide [8]. In a first case, hostile metabolic conditions (hypoxia, poor/intermittent nutrient bioavailability) can uncouple glycolysis, lipolysis and/or glutaminolysis from the tricarboxylic acid (TCA) cycle, resulting in defective cell respiration, a decrease in the mitochondrial membrane potential (Δψ) and, thus, an increased production of mitochondrial superoxide (mtO_2_^•−^) by the electron transport chain (ETC) [8,9,10,11]. In a second case, unbridled activity of the TCA cycle overloads the ETC with electrons, and electrons in excess leak out from the ETC to generate mtO_2_^•−^ [8]. When produced at high doses, superoxide, a reactive oxygen species (ROS) family member, can cause cell death [10]. However, when produced at subcytotoxic, intermediate doses in cancer cells, superoxide can activate prosurvival and prometastatic intracellular signaling pathways, including the transforming growth factor-β (TGF-β) pathway, thereby converting a cancer cell in a metastatic progenitor cell [8]. We thus considered mtO_2_^•−^ as the origin of a signal by which mitochondria, acting as metabolic sensors, instruct cancer cells to leave a hostile microenvironment. Consequently, we reasoned that metastatic prevention is an achievable therapeutic goal.

Several upstream events and downstream cascades could account for the prometastatic activities of mtO_2_^•−^, creating redundancies that would be difficult to drug collectively [12,13]. Therefore, we rather decided to directly target mtO_2_^•−^ itself by taking advantage of the existence of mitochondria-targeted antioxidants [14]. In proof-of-concept experiments, mitoTEMPO, a commercially available mitochondria-targeted antioxidant [15,16], was very efficient to prevent mouse B16F10 melanoma migration, invasion and metastasis in syngeneic mice [8]. However, this drug is in the public domain. Here, for future clinical applications, we decided to test mitoquinol mesylate (MitoQ), another mitochondria-targeted antioxidant [17] that was initially developed to decrease cell death caused by oxidative stress in pathologies such as Parkinson’s disease [18], Alzheimer’s disease (NCT03514875) and hepatitis C [19]. Compared to mitoTEMPO, MitoQ has already successfully passed Phase I safety clinical trials [20]. This history makes of MitoQ a potential first-in-class drug to prevent cancer metastasis. In a companion paper [21], we first verified that MitoQ was effective at inhibiting human breast cancer cell migration, invasion, clonogenicity, sphere formation and spheroid stability. Here, we further tested the capability of the drug to prevent metastasis in mice using human triple negative MDA-MB-231 cancer cell as a model, as well as spontaneous MMTV-PyMT mouse tumors. At clinically relevant doses, we report that MitoQ not only prevented breast cancer metastasis to the lungs, but also local recurrence after surgery, and that this treatment is compatible with chemotherapy.

## 2. Materials and Methods

### 2.1. Chemicals

Mitoquinol mesylate (MitoQ) was produced as previously described [17]. Doxorubicin (2 mg/mL), epirubicin (2 mg/mL), 5-fluorouracil (5-FU; 50 mg/mL), cisplatin (1 mg/mL), gemcitabine (38 mg/mL), paclitaxel (1 mg/mL) and cyclophosphamide (20 mg/mL) were kindly provided by the Central Pharmacy of the Cliniques Universitaires Saint-Luc (CUSL), Brussels, Belgium. Unless stated otherwise, all other chemicals were from Sigma-Aldrich (Overijse, Belgium). Equal volumes of solvent (DMSO) were used in control experiments.

### 2.2. MitoQ Biodistribution in Mice

MitoQ biodistribution was established using 8 weeks-old female BALB/cAnNCrl mice (Charles River, Beerse, Belgium) 4 h after the administration of a single dose of MitoQ (from 0 to 24 mg/kg) by oral gavage, which corresponds to the half-life of MitoQ in rat kidneys after oral delivery [22]. Animals were anesthetized using a 0.1 mL/20 g intraperitoneal injection of ketamine:xylazine (87.5 mg/kg:12.5 mg/kg), and sacrificed by cervical dislocation. Lungs, liver, heart and kidneys were collected, weighted and snap-frozen in liquid nitrogen. Organic extraction and MitoQ quantification using LC/MS/MS were performed as previously described [23]. Deuterated MitoQ (d_3_-MitoQ) was used as an internal standard.

### 2.3. Cells and Cell Culture

Triple-negative MDA-MB-231 human breast adenocarcinoma cancer cells were from Caliper (Mechelen, Belgium; catalogue #119369). HER2+ SkBr3 human breast adenocarcinoma cancer cells (catalogue #HTB-30) and triple-negative MDA-MB-436 human breast adenocarcinoma cancer cells (catalogue #HTB-130) were from ATCC (Manassas, VA, USA). All cancer cell lines were originally derived from pleural effusions [24,25,26]. MDA-MB-231 and SkBr3 cells were routinely cultured in DMEM containing 4.5 g/L glucose and GlutaMax (Thermofisher, Erembodegem, Belgium; catalogue #10566016) with 10% FBS, and MDA-MB-436 cells in IMDM containing GlutaMax (Thermofisher; catalogue #31980030) with 20% FBS. All cells were maintained at a subconfluent state in a humidified atmosphere with 95% room air, 5% CO_2_ at 37 °C. Cell authenticities were routinely verified with a short tandem repeat (STR) test (Eurofins Genomics, Ebersberg, Germany).

### 2.4. Experimental Metastasis Assay

For metastasis take assays, cancer cells were pretreated ± MitoQ for 6 h, and 10^6^ viable cells were injected into the tail vein of 5 weeks-old female Rj:NMRI-Foxn1 nu/nu mice (Janvier, Le Genest-Saint-Isle, France). After 4 weeks, mice were sacrificed and the lungs insufflated with 3 mL of Indian ink (15% in ddH_2_O). Mouse lungs were removed, washed with PBS and incubated overnight in Fekete’s solution (700 mL/L 100% ethanol, 32 mL/L 37% formalin, 40 mL/L glacial acetic acid, and ddH_2_O to reach 1 L). The next morning, lungs were transferred to a 70% ethanol solution and were further examined under a Stemi 2000-C dissection stereomicroscope (Zeiss, Zaventem, Belgium) to count the number of metastasis per lung.

### 2.5. Orthotopic Metastasis Assay

For spontaneous metastasis assays, MDA-MB-231 cells were prepared in medium containing 10% growth factor-reduced Matrigel (Corning, Tewksbury, MA, USA; catalogue #734-11-01). Five weeks-old female Rj:NMRI-Foxn1 nu/nu mice (Janvier) were injected with 10^6^ cancer cells in 100 µL of solution in the left second mammary fat pad. After tumor take (72 h), mice were daily treated ± 20 mg/kg MitoQ by oral gavage [20]. Tumor size was measured once per week with an electronic caliper. Four weeks after treatment initiation, primary tumors were surgically removed. Tumor recurrence was monitored until recurred tumors in the vehicle group reached about 200 cm^3^, i.e., 24 days after surgery. At this time point, mice were sacrificed and their lungs were collected and fixed in paraformaldehyde (PFA) 4% for immunocytochemistry.

### 2.6. Cytotoxicity Assay

To test the effects of combination treatments on cell numbers, MDA-MB-231 and SkBr3 cancer cells were plated in 96-well plates and treated with increasing concentrations of chemotherapeutic drug ± MitoQ for 24 h (epirubicin), 48 h (doxorubicin, 5-FU, cisplatin, paclitaxel) or 72 h (gemcitabine). Treatment times were adapted to known drug activities [27,28,29,30,31,32]. Cells were then fixed in 4% PFA and stained with crystal violet. The dye retained by the cells was solubilized in 10% acetic acid, and the optical density (570 nm) was measured using a SpectraMax i3 spectrophotometer equipped with a MiniMax imaging cytometer (Molecular Devices, Munich, Germany).

### 2.7. Spontaneous Tumor and Metastasis Model

Female FVB/N-Tg (MMTV-PyMT)634 Mul/J (MMTV-PyMT) mice that express the polyomavirus middle T antigen spontaneously develop orthotropic multifocal breast adenocarcinomas that spontaneously disseminate metastases to the lungs during mouse lifetime [33]. For spontaneous metastasis assays, female MMTV-PyMT mice (The Jackson Laboratory, Sacramento, CA, USA) were daily observed for signs of spontaneous primary tumors starting at 6 weeks of age. Once tumors became palpable, we waited 4 additional weeks without treatment to mimic a clinical time frame between first tumor signs and treatment initiation. Ten weeks-old mice with primary tumors of ~0.8 cm in diameter received a single dose of FEC chemotherapy (100 mg/kg 5-FU, 5 mg/kg epirubicin, 100 mg/kg cyclophosphamide), a clinically relevant neoadjuvant or adjuvant chemotherapeutic combination [34]. Animals were then randomly assigned to a group receiving 18 mg/kg MitoQ daily or an equal volume of vehicle by oral gavage. They were sacrificed at 16 weeks-old, and the lungs insufflated with Indian ink for metastasis quantification as described above.

### 2.8. Immunohistochemistry

Collected lungs were embedded in paraffin, sectioned at 5 µm-width, and immunostained using a primary rabbit recombinant anti-cytokeratin 19 (CK19) antibody (Abcam, Cambridge, UK; catalogue #ab76539) and a secondary Envision anti-rabbit antibody coupled to HRP (Dako; catalogue #K4003). Slices were counter-stained with hematoxylin. Images of whole lung slices were acquired on a SCN400 Slide Scanner (Leica Biosystems, Diegem, Belgium) and analyzed with the QuPath Software version 0.1.2 (Belfast, UK). Quantification was performed according to Chang and Erler [35], and the number of lung metastases was proportional to the positive areas of CK19 staining.

### 2.9. Statistics

All results are expressed as means ± standard error of the mean (SEM) for *n* independent observations. Error bars are sometimes smaller than symbols. Outliers were identified using Dixon’s Q test. Data were analyzed using GraphPad Prism 8.4.3 (San Diego, CA, USA). Student’s *t* test, Mann Whitney test, and two-way ANOVA were used where appropriate. A Log-Rank (Mantel-Cox) test was used to analyze the Kaplan-Meier graph. *p* < 0.05 was considered to be statistically significant.

## 3. Results

### 3.1. After Oral Delivery, MitoQ Accumulates in Mouse Organs

**Figure 1:**
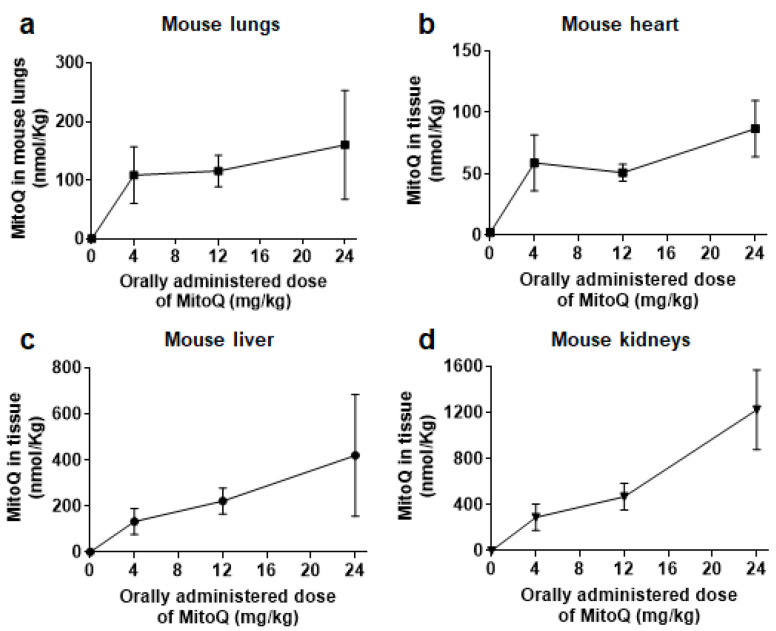
MitoQ biodistribution in mouse tissues. Female BALB/c mice received increasing doses of MitoQ per os, and tissues were collected 4 h later for analysis using LC/MS/MS. (**a**) MitoQ molality in function of the administered dose of MitoQ in mouse lungs (*n* = 3–4), (**b**) as in a, but in mouse heart (*n* = 5). (**c**) As in a, but in mouse liver (*n* = 3–6). (**d**) As in a, but in mouse kidneys (*n* = 3–5). All data are shown as means ± SEM.

Four hours after oral delivery (which corresponds to the half-life of MitoQ in rodents) [22,36], we previously reported that free MitoQ reached ~50 nM in mouse plasma for doses ranging from 12 to 24 mg/kg [21]. For comparison, in humans, oral dosing at 1 mg/kg resulted in a maximal plasma concentration of ~50 nM of MitoQ 4 h after delivery [20]. Since MitoQ accumulates in tissues (especially in mitochondria where it concentrates up to 100-fold) [37], we further determined MitoQ biodistribution in mice. At 4 h after the oral delivery of a single dose, MitoQ molality was slightly above 100 nmol/kg in mouse lungs for doses ranging from 4 to 24 mg/kg (Figure 1a), between ~50 and ~100 nmol/kg at the same doses in mouse heart (Figure 1b), and up to ~400 nmol/kg and ~1200 nmol/kg at an administered dose of 24 mg/kg in mouse liver and kidneys, respectively (Figure 1c,d). Based on the previous in vitro findings that MitoQ represses breast cancer cell migration, invasion and clonogenicity at extracellular concentrations ranging from 100 to 500 nM [21], we considered that concentrations of MitoQ compatible with its antimetastatic effects can be reached in mice upon oral delivery.

### 3.2. MitoQ Inhibits the Metastatic Take of Human Breast Cancer Cells in Mice

**Figure 2:**
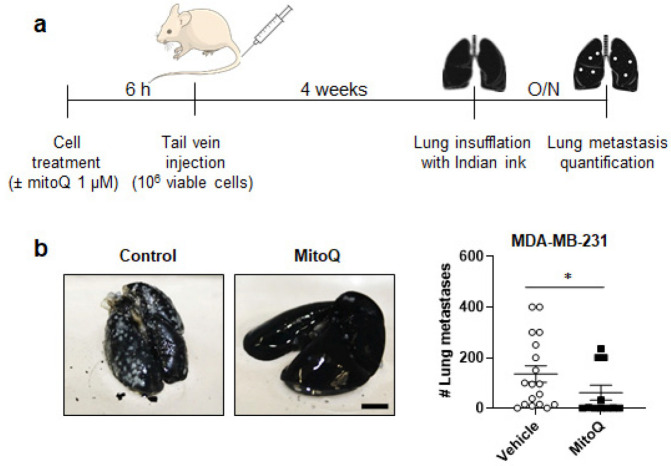
MitoQ inhibits the metastatic take of triple-negative human MDA-MB-231 breast cancer in mice. (**a**) Experimental protocol for tumor take assays where breast cancer cells were pretreated ± 1 µM MitoQ for 6 h before tail vein injection of 10^6^ viable cells in female NMRI nude mice. (**b**) Metastatic take of MDA-MB-231 human breast cancer cells in the lungs of mice using the protocol depicted in **a**. Left pictures are representative of lungs insufflated with Indian ink, where metastases appear as white dots quantified in the right graph (vehicle group *n* = 18; MitoQ group *n* = 11). Bar = 0.5 cm. All data are shown as means ± SEM. * *p* < 0.05 compared to vehicle, by Mann-Whitney test (**b**).

Among tested MDA-MB-231, MDA-MB-436 and SkBr3 human breast cancer cell lines, only MDA-MB-231 generated metastases in immunodeficient mice. We therefore proceeded with metastatic take and spontaneous metastasis assays with this cell line, and confirmed all results using the MMTV-PyMT mouse model of spontaneous breast cancer, known to spontaneously metastasize to the lungs [33]. In experimental lung metastasis assays, MDA-MB-231 cells were pretreated for 6 h ± 1 µM MitoQ before a tail vein injection of 10^6^ viable cells (note that, compared to in vitro experiments, we used a relatively high dose of MitoQ here to take into account dilution in the blood) (Figure 2a). Four weeks later, mice that had received MitoQ-treated cells developed significantly less metastases than mice that had received vehicle-treated cells (Figure 2b). In the control group, 88.9% of mice (16/18) presented >2 lung metastases and 11.1% (2/18) were lung metastasis-free at necropsy. In the MitoQ group, 36.4% of mice (4/11) were metastasis-free, 27.2% (3/11) had ≤2 lung metastases, and 36.4% (4/11) had >2 lung metastases. At this time point, we did not detect metastases in other organs.

### 3.3. MitoQ Inhibits Primary Tumor Recurrence and the Spontaneous Metastasis of Orthotopic MDA-MB-231 Tumors in Mice

**Figure 3:**
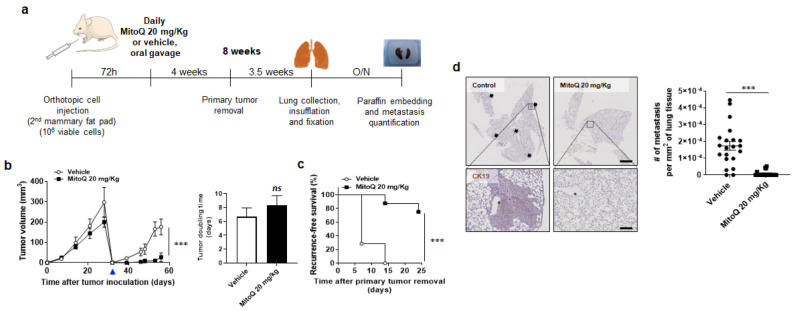
MitoQ prevents primary tumor recurrence and the metastatic dissemination of orthotopic triple-negative human MDA-MB-231 breast cancer in mice. (**a**) Experimental protocol for spontaneous MDA-MB-231 metastasis after orthotopic injection in the mammary fat pad of female NMRI nude mice. (**b**) On the left, primary tumor growth in mice using the protocol depicted in a (*n* = 10 per group until the day of surgery; *n* = 7 in the vehicle-treated group and *n* = 8 in the MitoQ-treated group after surgery). The blue arrow indicates the day of primary tumor resection. On the right, primary tumor doubling times from 100 to 200 mm^3^ before surgery (*n* = 10 per group). (**c**) Kaplan-Meier graph showing recurrence-free mouse survival after surgery. Data are expressed as percentage of mice per treatment group (*n* = 7 for vehicle and *n* = 8 for MitoQ). (**d**) At the end of the protocol shown in a, mouse lungs were removed, sliced, stained for cytokeratin 19 (CK19), counterstained with hematoxylin, and analyzed for the presence of metastases. Representative pictures are on the left at two different magnifications (bars = 16 mm for the two pictures on the top and 1 mm for the two pictures on the bottom), and metastasis quantification is on the right. All data are shown as means ± SEM. *** *p* < 0.001, *ns*: *p* > 0.05 compared to vehicle; by 2-way ANOVA (**b** left), Student *t* test (**b** right), Log-Rank (Mantel-Cox) test (**c**), or Mann-Whitney test (**d**).

As a pretreatment with 1 µM MitoQ might have triggered a delayed breast cancer cell death that we did not detect at the time of injection, it was important to confirm our data in spontaneous metastatic assays using an orthotopic approach. In these assays, we administered MitoQ daily using a dose included in the concentration range at which MitoQ accumulates in mouse organs (18–20 mg/kg) (Figure 3a). Assaying spontaneous metastasis involved a time lapse of 3 days between orthotropic implantation of MDA-MB-231 cells in the mammary fat pad of female mice and the beginning of treatment in order not to interfere with primary tumor take, and primary tumor removal at day +32 to unravel metastatic growth. MitoQ did not inhibit MDA-MB-231 primary tumor growth (Figure 3b), but largely prevented local tumor recurrence (Figure 3b,c). After surgery, primary tumors relapsed in 100% (7/7) of vehicle-treated mice, but only in 25% (2/8) of MitoQ-treated mice, and those tumors regrew significantly slower (Figure 3b). MitoQ thus significantly improved recurrence-free survival (Figure 3c). After mouse sacrifice on day +56, lung inspection using microscopy revealed that MitoQ had highly significantly prevented metastatic dissemination (Figure 3d). In the MitoQ group, 78.9% (15/19) of mice had no metastasis at all, whereas only 9.5% (2/21) were metastasis-free in the control group. All micro- and macrometastases were taken into account.

### 3.4. In Vitro, MitoQ Does Not Interfere with Chemotherapy-Induced Breast Cancer Cell Killing

**Figure 4:**
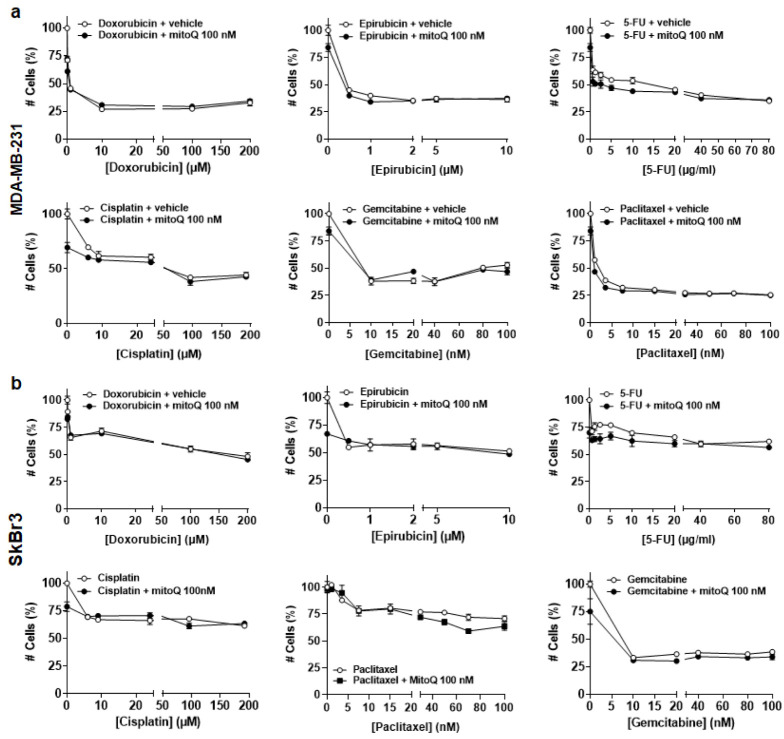
In vitro, MitoQ does not interfere with conventional chemotherapies used to treat breast cancer. (**a**) Counting of MDA-MB-231 cancer cells treated with increasing doses of chemotherapy ± MitoQ. Treatments were: 48 h with doxorubicin ± 100 nM MitoQ (*n* = 3), 24 h with epirubicin ± 100 nM MitoQ (*n* = 3), 48 h with 5-fluorouracil (5-FU) ± 100 nM MitoQ (*n* = 3), 48 h with cisplatin ± 100 nM MitoQ (*n* = 3), 72 h with gemcitabine ± 100 nM MitoQ (*n* = 3), and 48 h with paclitaxel ± 100 nM MitoQ (*n* = 3). (**b**) As in a, but using SkBr3 cancer cells (*n* = 3–4). All data are shown as means ± SEM. All *p* values are > 0.05; by 2-way ANOVA with Tukey’s post-hoc test (**a**,**b**).

Several types of chemotherapies act by increasing ROS production in cancer cells, which could participate in their anticancer effects [38]. To assess whether the antioxidant activity of MitoQ interfered with these processes, we counted cells treated in vitro with chemotherapy ± 100 nM MitoQ. Treatment times were adapted to known drug activities [27,28,29,30,31,32]. In MDA-MB-231 cancer cells, MitoQ did not alter the cytostatic/cytotoxic effects of increasing doses of doxorubicin, epirubicin, 5-fluorouracil (5-FU), cisplatin, gemcitabine or paclitaxel (Figure 4a). This absence of interference was confirmed using human HER2+ SkBr3 breast cancer cells (Figure 4b). Of note, IC_50′_s were not always reached, indicating intrinsic polyresistance of advanced cancer cell lines to chemotherapies. Cyclophosphamide was not tested in vitro, as it necessitates hepatic activation [39], so we tested it directly in MMTV-PyMT mice.

### 3.5. MitoQ Prevents the Metastatic Dissemination to the Lungs of Spontaneously Metastatic Breast Tumors in Mice

**Figure 5:**
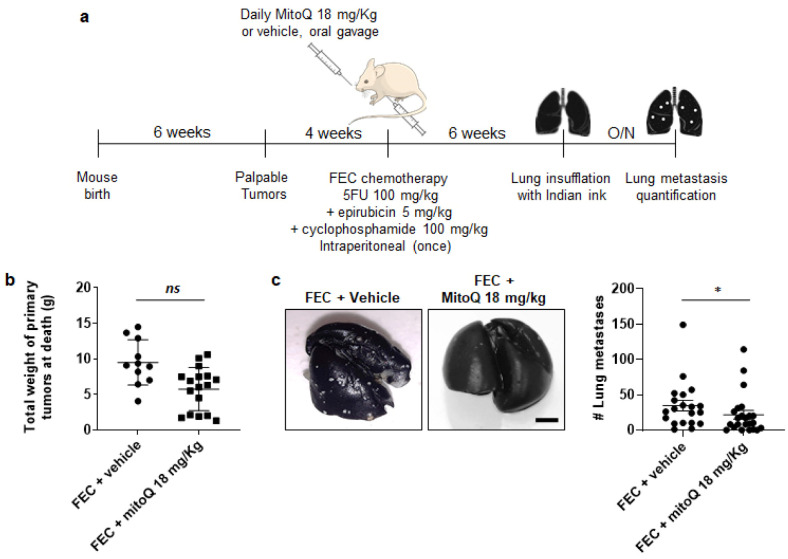
MitoQ prevents lung metastasis in spontaneous metastatic breast cancer in mice. (**a**) Experimental protocol for female MMTV-PyMT mouse treatment. (**b**) Total weight of primary breast tumors collected on the day of sacrifice of MMTV-PyMT mice treated as depicted in a (*n* = 11–17). (**c**) Lung metastases in MMTV-PyMT mice treated as depicted in a. Left pictures are representative of lungs insufflated with Indian ink, where metastases appear as white dots quantified in the right graph (*n* = 20–23). Bar = 0.5 cm. All data are shown as means ± SEM. * *p* < 0.05, *ns*: *p* > 0.05 compared to vehicle; by Mann-Whitney test (**b**,**c**).

We validated the antimetastatic activity of MitoQ in the MMTV-PyMT mouse model of spontaneous breast cancer, known to spontaneously metastasize to the lungs [33]. Hemizygous female MMTV-PyMT mice were treated as shown in Figure 5a, using FEC chemotherapy (5-FU 100 mg/kg, epirubicin 5 mg/kg, cyclophosphamide 100 mg/kg) as a standard of care [34] ± a daily oral administration of MitoQ (18 mg/kg). After six weeks of treatment, total primary tumor weight (several tumors per animal) was similar in animals having received FEC + vehicle versus FEC + MitoQ (Figure 5b), indicating no interference of MitoQ with FEC chemotherapy. However, MitoQ significantly repressed the number of lung metastases (Figure 5c). In the FEC + vehicle group, all mice (20/20) had metastasis with a median number of 28 lesions per mouse, whereas 4 of 23 mice were metastasis-free in the FEC + MitoQ group with a median number of 10. Of note, MitoQ could not be tested independently of chemotherapy in this model since surgical removal of multifocal, highly invasive breast tumors was not possible, and untreated mice died before metastatic dissemination.

## 4. Discussion

To our knowledge, no specific anticancer treatment exists to date that prevents metastatic dissemination, making it a clinical priority [40]. Here, we report that MitoQ, a mitochondria-targeted antioxidant [20], prevents human breast cancer recurrence and metastasis in mice.

MitoQ has a number of properties that make it a potential chronic anticancer treatment. These include target selectivity for mtO_2_^•−^, oral bioavailability, a long time of action (as it accumulates in mitochondria), and limited toxicity in humans (some nausea and vomiting reported at the maximal tolerated oral dose [MTD] of 1 mg/kg) [17,18,20,37,41]. This study adds preclinical efficacy over a range of doses achievable in humans, and compatibility with chemotherapy. MitoQ has already undergone Phase I safety and Phase II efficacy clinical trials for pathologies other than cancer [18,19,20], and we propose this molecule as a drug candidate for the clinical prevention of breast cancer recurrence and metastasis.

Our working hypothesis that mitochondria are metabolic sensors promoting metastasis was initially based on two theories. First, a current paradigm is that metastatic progenitor cells originate from tumor areas where a shortage of oxygen and nutrients and extracellular acidification set a selection pressure on cancer cells, generating cancer cells that are able to leave metabolically hostile microenvironments [42]. Hypoxia is thought to increase mtROS production, which activates prometastatic pathways [43]. Second, experimental manipulations that trigger subcytotoxic mtROS production in cancer cells were shown to promote migration, invasion and metastasis [7,8], and a transfer of mitochondria can also transfer the metastatic phenotype [7]. Our study goes beyond these artificial models, as we used naturally metastatic human breast cancer cells and the MMTV-PyMT mouse model to show that mtROS are a key contributor to cancer metastasis.

MitoQ not only prevented metastasis but also the local recurrence of human breast cancer in mice after surgery. This was unexpected, and could be explained by a common dependency of metastases and recurrences on “stemness”. Two experimental observations support this proposition. First, at clinically relevant doses ranging from 100 nM to 500 nM, MitoQ largely blocked clonogenicity, sphere formation and spheroid stability, while cancer cell proliferation and primary tumor growth were barely affected [21]. It also decreased the expression of stemness-related genes in spheres [21]. Second, MitoQ repressed breast cancer cell respiration, and a high oxygen consumption rate (OCR) is a fundamental characteristic of proliferating breast cancer stem cells [44]. Thus, upon chronic treatment, a loss of stem cell characteristics would account for the preventive effects of MitoQ on metastasis and recurrence, whereas the development of pre-established micrometastases would not be affected. Still, in that case, we believe that decreasing metastatic burden would already be an important clinical achievement. At best, preventing or delaying metastasis and/or local recurrence in breast cancer could increase cure rates with conventional treatments, while also enhancing the time window before the exhaustion of treatment lines.

While we have no evidence for the development of resistance to MitoQ, it is of course possible that cancer cells will find ways to counteract its effects. These could include enhanced export of the molecule from the cell or enhanced mtROS production to overcome the action of MitoQ. As MitoQ is rapidly accumulated within mitochondria, it is shielded from many potential mechanisms of resistance [17], but only time will tell if new pathways will emerge.

Comparatively to MitoQ, general antioxidants that interfere with many redox reactions in cells have unpredictable effects and should be avoided to treat human cancers [13]. Compared to these, MitoQ is expected to be safer as it selectively targets mtROS [41]. Importantly, after a single oral dose, the MTD (1 mg/kg/day) used in human trials yielded a similar ~50 nM maximal plasma concentration [20] as in our mouse models where MitoQ showed clear anticancer effects [21]. As for mice in this report, human tissue levels of MitoQ are expected to be higher than in the plasma due to uptake into cells and accumulation within mitochondria. Repeated administration, which can be anticipated for recurrence and metastasis prevention, is expected to further increase these levels. We therefore consider our data to be clinically relevant.

Other antioxidants selectively targeting mitochondria have been developed (ea., thiobutyltriphenylphosphonium bromide, mitoVitE, mitoTEMPO, SkQ1 …), but their mode of action and selectivity for mtO_2_^•−^ versus other mtROS or mitochondrial nitrogen species are by far less well established [45,46]. Together with oral bioavailability and intramitochondrial accumulation, we believe that the ability of MitoQ to regenerate itself after interaction with mtROS [20], instead of being destroyed as it is the case for most other mitochondria-targeted antioxidants, is an advantage for chronic treatment. We further established the in vitro compatibility of MitoQ with the major types of chemotherapies currently used for breast cancer treatment. While some previous reports suggested that several chemotherapeutic agents induce ROS production to kill cancer cells (see reference [38] for a review), other reports [47,48] and our data rather indicate that such response of cancer cells probably represents a rescue strategy to activate general redox defenses (mitohormesis) and/or a tendency to reactivate the metastatic program.

## 5. Conclusions

In conclusion, our preclinical evaluation suggests that MitoQ is a first-in-class drug candidate to prevent human breast cancer metastasis and recurrence under conditions that are compatible with human therapy. Follow-up preclinical studies are now warranted to test the effects of MitoQ at other metastatic sites and in other types of metastatic cancers.

## Data Availability

All data are contained within the article.
